# Western Australian medical students’ attitudes towards artificial intelligence in healthcare

**DOI:** 10.1371/journal.pone.0290642

**Published:** 2023-08-31

**Authors:** Jonathon Stewart, Juan Lu, Nestor Gahungu, Adrian Goudie, P. Gerry Fegan, Mohammed Bennamoun, Peter Sprivulis, Girish Dwivedi

**Affiliations:** 1 School of Medicine, The University of Western Australia, Crawley, Western Australia, Australia; 2 Harry Perkins Institute of Medical Research, Murdoch, Western Australia, Australia; 3 Department of Computer Science and Software Engineering, The University of Western Australia, Crawley, Western Australia, Australia; 4 Department of Cardiology, Fiona Stanley Hospital, Murdoch, Western Australia, Australia; 5 Department of Emergency Medicine, Fiona Stanley Hospital, Murdoch, Western Australia, Australia; 6 Department of Endocrinology and Diabetes, Fiona Stanley Hospital, Murdoch, Western Australia, Australia; 7 Medical School, Curtin University, Bentley, Western Australia, Australia; 8 Western Australia Department of Health, East Perth, Western Australia, Australia; The University of Alabama, UNITED STATES

## Abstract

**Introduction:**

Surveys conducted internationally have found widespread interest in artificial intelligence (AI) amongst medical students. No similar surveys have been conducted in Western Australia (WA) and it is not known how medical students in WA feel about the use of AI in healthcare or their understanding of AI. We aim to assess WA medical students’ attitudes towards AI in general, AI in healthcare, and the inclusion of AI education in the medical curriculum.

**Methods:**

A digital survey instrument was developed based on a review of available literature and consultation with subject matter experts. The survey was piloted with a group of medical students and refined based on their feedback. We then sent this anonymous digital survey to all medical students in WA (approximately 1539 students). Responses were open from the 7^th^ of September 2021 to the 7^th^ of November 2021. Students’ categorical responses were qualitatively analysed, and free text comments from the survey were qualitatively analysed using open coding techniques.

**Results:**

Overall, 134 students answered one or more questions (8.9% response rate). The majority of students (82.0%) were 20–29 years old, studying medicine as a postgraduate degree (77.6%), and had started clinical rotations (62.7%). Students were interested in AI (82.6%), self-reported having a basic understanding of AI (84.8%), but few agreed that they had an understanding of the basic computational principles of AI (33.3%) or the limitations of AI (46.2%). Most students (87.5%) had not received teaching in AI. The majority of students (58.6%) agreed that AI should be part of medical training and most (72.7%) wanted more teaching focusing on AI in medicine. Medical students appeared optimistic regarding the role of AI in medicine, with most (74.4%) agreeing with the statement that AI will improve medicine in general. The majority (56.6%) of medical students were not concerned about the impact of AI on their job security as a doctor. Students selected radiology (72.6%), pathology (58.2%), and medical administration (44.8%) as the specialties most likely to be impacted by AI, and psychiatry (61.2%), palliative care (48.5%), and obstetrics and gynaecology (41.0%) as the specialties least likely to be impacted by AI. Qualitative analysis of free text comments identified the use of AI as a tool, and that doctors will not be replaced as common themes.

**Conclusion:**

Medical students in WA appear to be interested in AI. However, they have not received education about AI and do not feel they understand its basic computational principles or limitations. AI appears to be a current deficit in the medical curriculum in WA, and most students surveyed were supportive of its introduction. These results are consistent with previous surveys conducted internationally.

## Introduction

Artificial Intelligence (AI) broadly refers to computer systems that can perform tasks that usually require human intelligence [1]. There exists both optimism and excitement over the potential for AI to improve society, and valid apprehension and concerns over its potential failings or misuse [2]. Large surveys have been undertaken to assess public attitudes towards AI in Australia and internationally [1, 2]. Healthcare has been consistently identified as one of the most promising areas for the application of AI, and there is strong public support for the use of AI in healthcare [1, 3].

The integration of AI based tools into healthcare has begun, and some specialties are likely to be more disrupted than others [4]. It has been recommended that AI applications should be developed in close collaboration with practicing clinicians [5]. Future clinicians will likely be required to become more familiar with specific AI techniques such as machine learning (ML) and deep learning (DL), understanding not only their potential applications but also their limitations, and be able to identify when the AI algorithms have made a mistake [6]. This requires specialised training and is likely to lead to an increasing need for AI education as part of general medical education [6]. There are now calls for medical students to be educated about AI [6–9]. The attitudes of medical students towards AI in medicine has been investigated internationally [6]. Pinto Dos Santos et al. assessed attitudes of 263 medical students at three major universities in Germany towards AI in medicine, with a focus on AI in radiology [10]. They found that 52% of respondents were aware of ongoing discussions regarding AI in radiology, however 68% stated that they were unaware of the technologies involved. The majority of respondents agreed with the statements that AI will revolutionise radiology and medicine in general and agreed that AI should be part of medical training. Sit et al. assessed attitudes of 484 medical students from 19 medical school in the United Kingdom regarding AI in medicine [11]. The majority of respondents felt that AI will play an important role in healthcare, that teaching in AI would be beneficial for their careers, and that students should receive training in AI as part of their medical degree. Less than 10% of students had received any teaching on AI, and most students have acquired their knowledge of AI through mass media. Cho et al. surveyed 100 students from the Seoul National University College of Medicine regarding their attitudes towards AI in dermatology [12]. They reported a discrepancy between students’ high levels of interest in AI and the lack of formal education in AI in medical training, with 83% of students agreeing with the statement that AI education is necessary for the medical school curriculum. A recent systematic review by Mousavi Baigi et al. assessed results from 38 studies of healthcare students’ attitudes towards AI, concluding that the majority of students have a positive attitude towards AI, however often had a low knowledge of AI, and limited skills in working with AI [13]. Few studies have empirically tested medical students AI knowledge. An exception is Blacketer et al., who conducted a voluntary online formative examination of 245 medical students from three centres internationally [14]. Medical students performed poorly on questions that focused on interpreting and critically appraising ML research, however performed well on questions on interpreting conclusions and statistical significance of ML research. In a post-examination survey, medical students indicated that they were interested in ML and 79.3% felt they would be required to interact with ML application throughout their careers. However, 50% answered that they had no previous exposure to these technologies. In summary, surveys conducted internationally have found widespread interest in AI amongst medical students, with medical students believing that knowledge of AI will be important in their future careers [13, 15]. Despite this, multiple surveys have found medical students self-report a limited understanding of AI, and have had limited exposure to AI, with most exposure coming from popular media [14–16]. Medical students internationally agree that AI training should be part of their medical education [10, 11, 15, 17]. However, there is currently a lack of formal AI training in medical education [15, 18]. A systematic review by Sapci et al. assessing the current state of AI training for medical students found that while there are recommendations to integrated AI into the medical curriculum, this has not yet occurred [6]. Of the few medical students who have received training in AI, almost all felt it was useful [11, 15].

Western Australia (WA) has a population of approximately 2.7 million people. There are three universities (The University of Western Australia, Notre Dame University, and Curtin University) offering either undergraduate or post-graduate medical degrees. No similar surveys have been conducted in WA and it is now known how medical students in WA feel about the use of AI in healthcare or their understanding of AI.

We conducted an anonymous voluntary cross-sectional digital survey (S1 Appendix) of medical students from all medical schools in WA to assess their attitudes, perceptions, and understanding towards AI in healthcare.

We aimed to

1. Assess WA medical students’ attitudes towards AI, both in general and in healthcare.
2. Assess WA medical students’ attitudes towards AI education in the medical curriculum.

## Methods

This study received ethics approval from The University of Western Australia (HREC 2021/ET000003) and Curtin University (HRE2021-0149), and approval as an external project from Notre Dame University. The survey instrument was developed based on a review of available literature and with input from specialists in emergency medicine, cardiology, clinical informatics, computer science, and artificial intelligence [10–12, 19, 20]. Our survey instrument includes questions previously developed and validated by Cho et al. (“I am interested in AI”), Sit et al. (“AI will play an important role in healthcare”, “Some specialties will be replaced by AI during my lifetime”, “I have an understanding of the basic computational principles of AI”, “At the end of my medical degree, I will have a better understanding of the methods used to assess medical AI algorithm performance. “, “Overall, at the end of my medical degree, I feel I will possess the knowledge needed to work with AI in routine clinical practice”, “I have received teaching/training in artificial intelligence”), and Pinto Dos Santos et al. (“Artificial intelligence will improve medicine in general”, “Artificial intelligence should be part of medical training”) [10–12]. The draft survey was then piloted with a small group of medical students and refined based on their feedback. Answers to these pilot questions were not included in final analysis. A recruitment email with a link to the anonymous digital survey (Qualtrics XM Survey Software) was sent to all undergraduate and post-graduate medical students in WA (approximately 1539 students). The survey was also posted on the universities’ online learning sites. A reminder email was sent at the discretion of participating medical schools. The survey was displayed to students following their informed consent. All students were shown the same questions in the same order. The survey was open from over a two-month time period from the 7th of September 2021 to the 7th of November 2021. Quantitative analysis was undertaken using Qualtrics®. Free text comments were exported to NVivo software (QSR International) for qualitative analysis. Comments were iteratively coded by two investigators (JS and JL) using open coding techniques until most comments could be classified under broad themes.

## Results

### Demographics

Over a 12-week period, 137 students viewed the digital participant information and consent form and consented to take part (8.9% response rate). Of these, 3 students did not answer any questions and 134 students answered one or more question. Student demographics are described in Table 1. The majority of students (82.0%) were 20–29 years old, were studying medicine as a postgraduate degree (77.6%), and had started clinical rotations (62.7%).

**Table 1:** Student demographics.

Demographics		
Age	Count (n = 134)	Percent
< 20	4	3%
20–29	110	82%
30–39	16	12%
40–49	3	2%
≥ 50	1	1%
**Gender**		
Male	72	54%
Female	59	44%
Non-binary	1	1%
Prefer not to say	2	1%
**Degree**		
Undergraduate	30	22%
Postgraduate	104	78%
**Started clinical rotations?**		
Yes	84	63%
No	50	37%

### AI interest and knowledge

**Fig 1:**
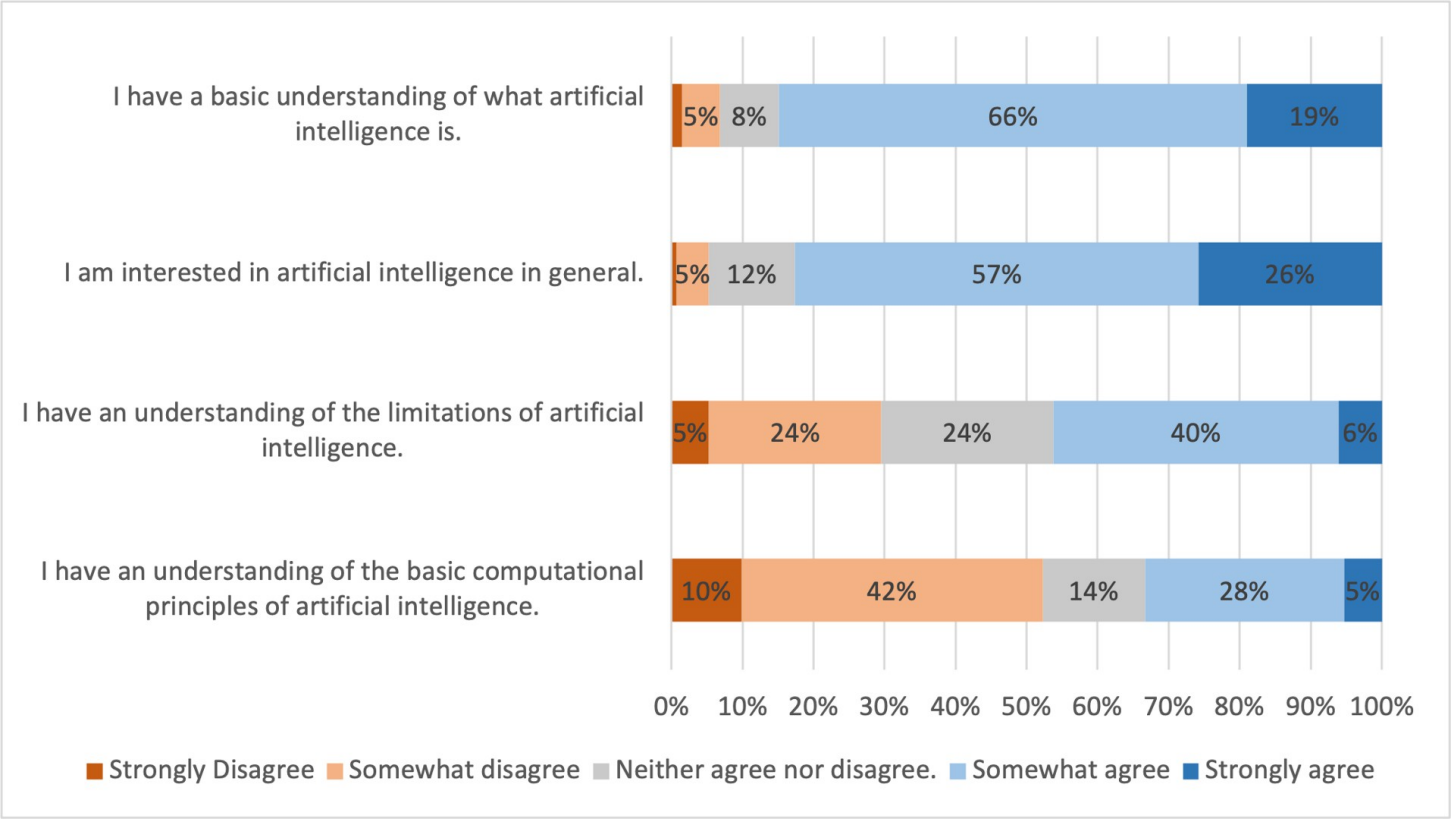
Medical students’ self-reported knowledge of artificial intelligence and interest in artificial intelligence. Percentages of students who strongly disagreed, somewhat disagreed, neither agreed nor disagreed, somewhat agreed, or strongly agreed with corresponding statements. Percentage ≤ 2 are not labelled in the figure to improve readability.

Students were interested in AI, and self-reported having a basic understanding of AI. The majority of students somewhat agreed or strongly agreed with the statement "I am interested in artificial intelligence in general" (82.6%) and the statement "I have a basic understanding of what artificial intelligence is" (84.8%) (Fig 1). Despite this, less than half (46.1%) of students somewhat agreed or strongly agreed with the statement "I have an understanding of the limitations of artificial intelligence". Over half (52.2%) of the students somewhat disagreed or strongly disagreed with the statement "I have an understanding of the basic computational principles of artificial intelligence." Students selected that they had heard of machine learning (59%), deep learning (47%), and Deepfakes (57%) (Table 2). Few students had heard of specific topics well known in the AI community such as convolutional neural networks (19%), ImageNet (10%), TensorFlow (8%), generative adversarial neural networks (6%) or GPT-3 (5%).

**Table 2:** Count and percent of students who selected that they had heard of topics related to artificial intelligence.

Topic	Count	Percent
Python	94	70%
Machine learning	79	59%
Deepfakes	77	57%
Deep learning	63	47%
OpenAI	53	40%
DeepMind	33	25%
Convolutional Neural Networks	25	19%
ImageNet	13	10%
TensorFlow	11	8%
Generative Adversarial Neural Networks	8	6%
GPT-3	7	5%
PyTorch	6	4%

### Education in AI

**Fig 2:**
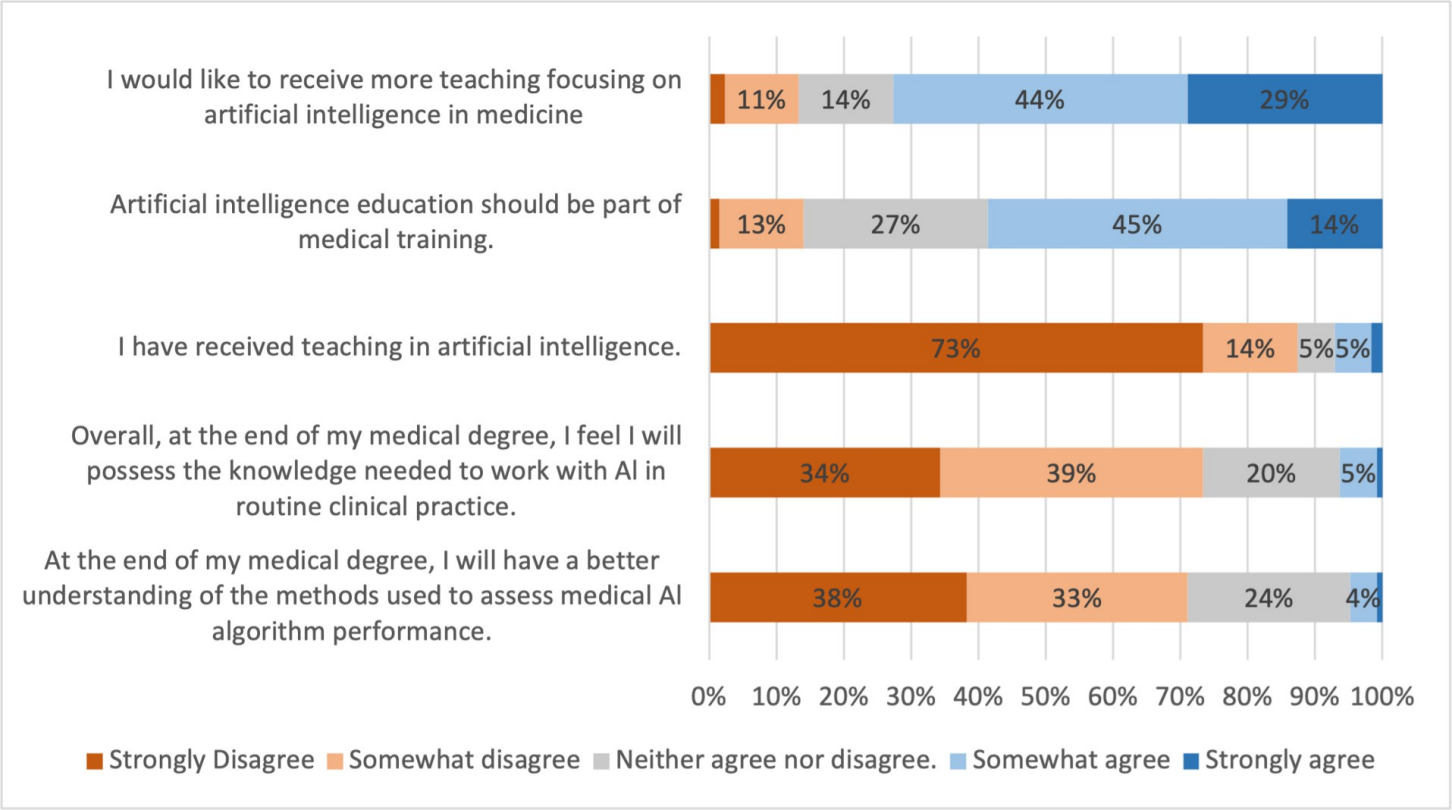
Medical students’ education in artificial intelligence and attitudes towards incorporation of artificial intelligence into the medical curriculum. Percentages of students who strongly disagreed, somewhat disagreed, neither agreed nor disagreed, somewhat agreed, or strongly agreed with corresponding statements. Percentage ≤ 2 are not labelled in the figure to improve readability.

The majority of students had not received formal education in AI, with 73.4% strongly disagreeing and a further 14.1% somewhat disagreeing with the statement "I have received teaching in artificial intelligence" (Fig 2). Students also strongly (34.4%) or somewhat (39.0%) disagreed with the statement "Overall, at the end of my medical degree, I feel I will possess the knowledge needed to work with Al in routine clinical practice." The majority (58.6%) of students, somewhat agreed or strongly agreed with the statement "Artificial intelligence education should be part of medical training" and 72.7% of students somewhat agreed or strongly agreed with the statement "I would like to receive more teaching focusing on artificial intelligence in medicine".

### AI in medicine

**Fig 3:**
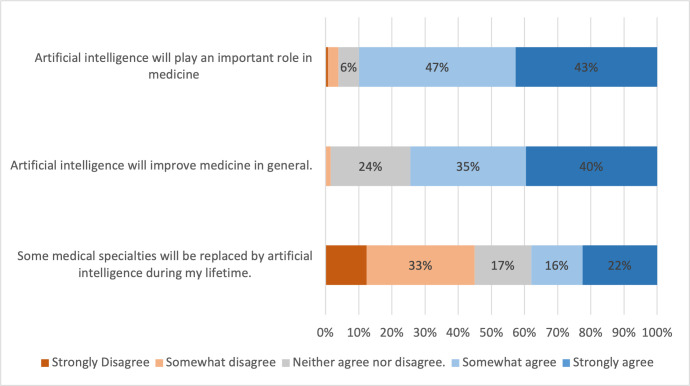
Medical students’ attitudes towards AI in medicine. Percentages of students who strongly disagreed, somewhat disagreed, neither agreed nor disagreed, somewhat agreed, or strongly agreed with corresponding statements. Percentage ≤ 2 are not labelled in the figure to improve readability.

Medical students appeared optimistic regarding the role of AI in medicine, with 89.9% somewhat or strongly agreeing with the statement "artificial intelligence will play an important role in medicine", and 74.3% somewhat agreeing or strongly agreeing with the statement "artificial intelligence will improve medicine in general" (Fig 3).

### AI in their future practice

**Fig 4:**
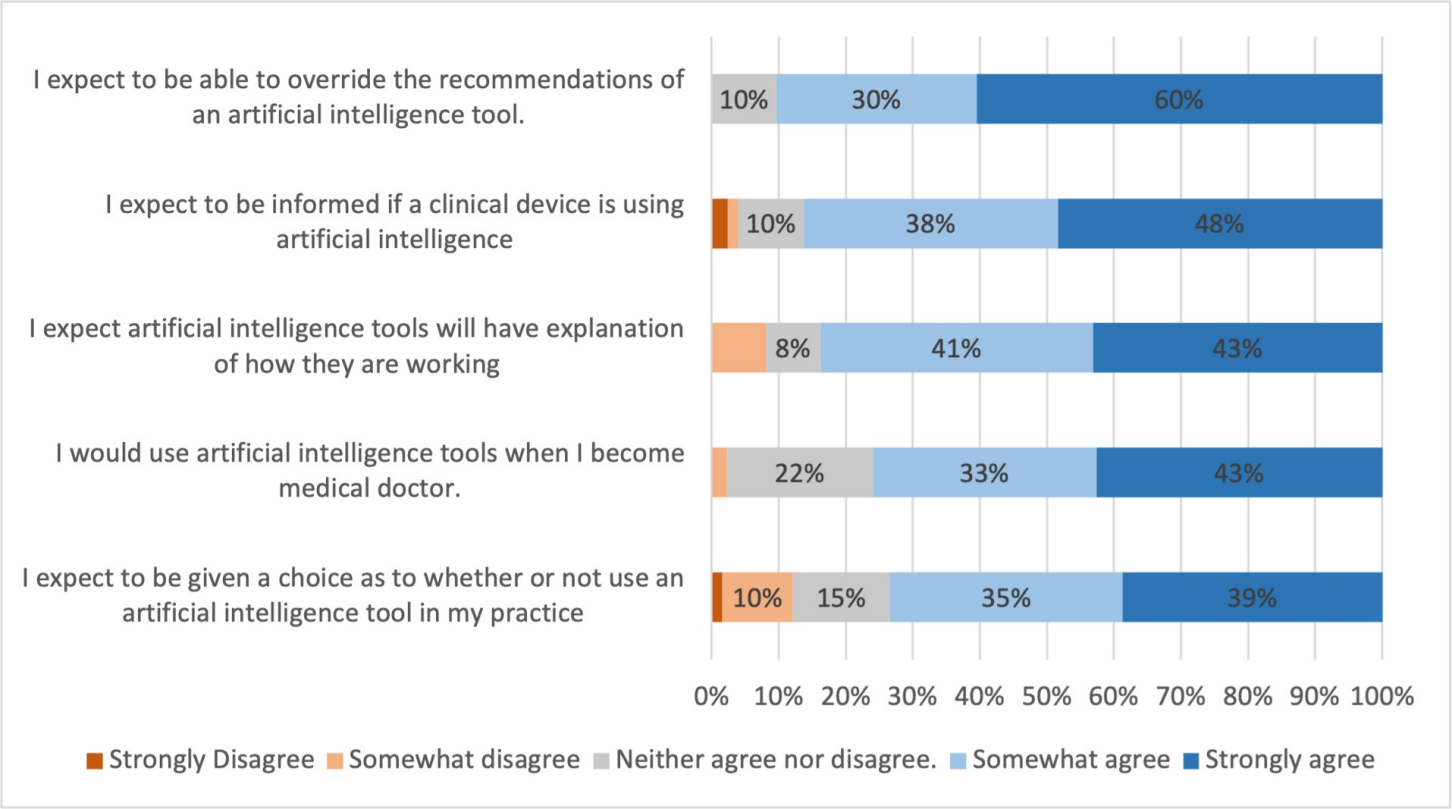
Medical students’ attitudes towards the use of AI in their future practice. Percentages of students who strongly disagreed, somewhat disagreed, neither agreed nor disagreed, somewhat agreed, or strongly agreed with corresponding statements. Percentage ≤ 2 are not labelled in the figure to improve readability.

Medical students expect to be informed if a clinical device is using AI (86.3% somewhat or strongly agree) (Fig 4). They also expect AI tools will have explanation of how they are working (83.7% somewhat or strongly agree), and expect to be given a choice as to whether or not they use AI tools in their practice (73.4% somewhat agreed or strongly agreed)

### AI and job security

The majority (56.6%) of medical students were either “not really concerned" (37.7%) or "not at all concerned" (18.9%) on the impact of developments in AI on their job security as a doctor (S1 Fig). Only 14.8% of students said they were either concerned or very concerned regarding job security. Students were split as to whether some medical specialties would be replaced by AI in their lifetime, with 45.0% of students somewhat disagreeing or strongly disagreeing and 38% somewhat agreeing or strongly agreeing that this would happen (Fig 3).

The specialties predicted to be most impacted by AI were radiology (selected by 72.6%), pathology (58.2%), medical administration (44.8%), surgery (35.1%) and anaesthesia (31.3%) (S1 Table). The specialties predicted to be least impacted by AI were psychiatry (selected by 61.2%), palliative care (48.5%), obstetrics and gynaecology (41.0%), general practice (36.6%), and paediatric medicine (36.6%) (S2 Table).

Most students were neither more nor less likely to consider a career in radiology given developments in AI (57.9%), however 33.9% of students were "less likely" or "much less likely". (S2 Fig) A minority of students (8.3%) were "more likely" or "much more likely" to choose radiology as a specialty given developments in AI.

### Qualitative analysis—Job security

Fifty-six students completed an optional free text submission explaining their answers regarding AI and job security. The most commonly occurring themes and representative comments are shown in Table 3. Medical students expressed that AI will be a useful tool (24/56) and act as an adjunct to clinical expertise.

**Table 3:** Thematic analysis of medical students’ free text explanations of how developments in artificial intelligence make them feel regarding job security as a doctor.

Theme	Count (percent)	Representative comments
Doctors won’t be replaced	37 (66.1%)	“Artificial intelligence won’t replace doctors. Instead, doctors who can use artificial intelligence will replace doctors who can’t use it. (Heard this somewhere but can’t remember where).”
		“I have an educational background in Data Science. I do not believe that the technology will replace the role of healthcare providers.”
		“I strongly believe that while AI has great potential, it will never (or not anytime soon) be ready to replace doctors or anyone in the health sector.”
AI as a tool	24 (42.9%)	“AI should be looked at as a tool to help reduce human error in medicine and to work alongside a Physician rather than independently of them.”
		“In the end, AI is just another tool.”
		“It will be integrated like many other tools”
		“I feel that AI will be utilized as a tool to aid medical practice, rather than replace it. It may mean everyday work will appear different.”
Non-technical skills	20 (35.7%)	“A doctor will still be needed to communicate with and educate patients and carry out tasks that may be suggested by artificial intelligence. I do think that the role of a doctor will change to be more of an educator and communicator”
		“Empathy and human touch matter to most people”
		“I think that AI will not apply to all specialties in medicine, and, more importantly, will not be able to out-perform human physicians on elements of medicine and healing that cannot be derived via AI. For example, ensuring the patient feels heard, human contact, etc.”
		“There will always be areas of medicine that require the human contact—empathy. Much more appreciable in a human compared to AI”
AI will have a significant impact.	8 (14.3%)	“Artificial intelligence has created huge changes In other industries I think it is unwise to think it magically won’t affect medicine in profound ways too. I don’t think it will completely push us out of a job but I am concerned about how medicine will change”
		“I know AI can have massive implications on assisting with multiple medical fields; studies in Africa showed that an AI was able to distinguish between normal and abnormal CXRs in 98% of images reviewed. This could imply cheaper surveillance strategies in occupational health.”
		“I like radiology, and would love to be a radiologist, but believe they are already obsolete
		“In my lifetime, developments in AI will certainly progress to the point where it can accurately and consistently diagnose conditions, given that it receives correct inputs like patient symptoms/signs. It has certainly achieved that in many other fields where the input is more clear cut (e.g. chess, online games, population simulations).”
Not concerned	7 (12.5%)	“Historically, technological improvements have not reduced the amount of doctors in the work force. Technological improvements are usually associated with increased specialisation and increased efficiency, but have not contributed to loss of jobs despite common misconception.”
		“Change is part of life, we just have to adapt to emerging technologies.”
		“I’m not afraid of change and I’m more than willing to learn to work with AI
Slow development	6 (10.7%)	“Within my lifetime I do not expect that AI will be sufficiently complex to replace one-to-one human medical care”
		“I don’t see AI progressing to the point of not requiring any human input any time soon.”
		“I am interested in a field of medicine (internal medicine/physician) which I don’t think artificial intelligence will ’replace’ over my lifetime.”
Concerned	4 (7.1%)	“It does concern me however that I have spent a long time studying and potentially will not have a job that I worked hard for.”
		“Partially concerned that they may affect future employment opportunities”
		“Why would you pay a human to do something if a machine can do it better and more efficiently. It should be a concern of every individual in the world. There is almost no governance over artificial intelligence and no one seems to care.”

A large number of students (37/56) believe that doctors will not be replaced by AI. Though some (8/56) remarked that AI would have a significant impact on medicine. Many medical students (20/56) wrote about non-technical skills that are important in the practice of medicine, such as empathy and communication skills, giving these as examples of things that AI will not be able to achieve. Some students (6/56) felt that AI development would be slow, and that AI would be unlikely to reach the level required to impact their job security within their lifetime and some (7/56) stated that they were not concerned by developments in AI. Conversely, a minority of students (4/56) voiced some level of concern regarding the impact of AI on medicine and their future job security.

### Qualitative analysis—Career in radiology

Forty-eight students completed an optional free text submission explaining whether developments in AI influence the likelihood of them considering a career in radiology. The most commonly occurring themes and representative comments are shown in Table 4. A large number of students (22/48) indicated that they were not interested in pursuing a career in radiology, regardless of developments in AI. Many students (14/48) expressed that AI would significantly impact radiology as field, with a smaller number (4/48) suggesting that AI will completely replace radiologists. Interventional radiology was identified as being less likely to be affected than diagnostic radiology (4/48). Other students expressed that AI would be a useful tool to radiologists (7/48), and that AI would improve radiology as a field and as a career choice (7/48). A minority of students (3/48) directly stated that radiologists would not be replaced by AI.

**Table 4:** Thematic analysis of medical students’ free text explanations of how likely they are to consider a career in radiology given developments in artificial intelligence.

Theme	Count (percent)	Representative comments
Not interested	22 (45.8%)	“Didn’t want to be a radiologist anyway so AI presence doesn’t bother me”
		“Don’t have a particular interest in either AI or radiology so this is unlikely to influence my decision”
		“I’m not interested in a career in radiology at all, regardless of AI.”
AI will have a significant impact.	14 (29.2%)	“I feel this field is the most immediately at risk from developments in AI, given the advancements in image recognition technology.”
		“Career prospects would decrease.”
		“Radiology will be strongly impacted by AI programs, and I don’t feel like it would be a very involving field anymore. Less hands on going forward in the future.”
AI will improve radiology.	7 (14.6%)	“I think that the ways in which AI will affect radiology will only ease the burden on an overtaxed resource (being the radiology department as a whole).”
		“If anything I think AI will just make a radiologists life at its base easier”
		“It’ll only make the field cooler.”
		“I think if anything it makes the job seem more interesting since the radiologist is more likely to take on a different role (ie assessing AI efficiency rather than repetitively interpreting scans).”
AI as a tool	7 (14.6%)	“An AI may prove very useful to a radiologist by either screening, or even clarifying an image that may be over/underexposed.”
		“I see AI as a useful supplement to radiology.”
		“Radiology is evolving with AI and functions as a supplement.”
Radiologists will be replaced	4 (8.3%)	“From what I know and have heard, it is one of the specialties that will be eventually replaced by AI as it comes down to pattern recognition without the need for as much interaction as some of the other parts of medicine.”
		“I believe AI will supersede this speciality within 20 years”
		“It is routine and automatable and will be done by robots in the future”
		“THEY WILL NOT EXIST IN 10 years. Machines already do it better.”

### Qualitative analysis—Further thoughts and comments

At the end of the survey, 19 students provided further thoughts and comments about AI in medicine. The most commonly occurring themes and representative comments are shown in Table 5. Students (5/19) suggested that AI should be a part of current medical training. One student reported that they had already begun undertaking self-directed learning. Non-technical skills were again mentioned (3/19), and students reiterated that AI should be used as a tool (4/19). Some students (3/19) were optimistic regarding the use of AI in medicine. Students also discussed risks (3/19) and referenced modern media or pop-culture (2/19).

**Table 5:** Thematic analysis of medical students’ free text further thoughts and comments about artificial intelligence in medicine, submitted at conclusion of the digital survey.

Theme	Count (percent)	Representative comments
Requested teaching	5 (26.3%)	“Absolutely need to be taught in med school starting asap”
		“Pls teach more in our course”
		“We should be taught the basics of AI in med school because there needs to be an intersection between AI and medicine, instead of having one person develop the AI and another person to understand the human body.”
Discussed risks	3 (15.8%)	“I think a downside will be the non-medically educated "google/self-taught experts" claiming an understanding that they don’t possess based on the results of an AI determination, but that is not hugely different from what happens already.”
		“Lawyer-up. Major ethical and legal issues will arise if (and when) mistakes are made using AI as a guide.”
		“MORE RULES AND REGULATIONS ARE NEEDED.”
AI as a tool.	4 (21.1%)	“It should be developed as a tool for use by humans, not to replace them.”
		“I expect AI to become a part of medicine, but should be a tool to use alongside clinical judgement and human decision-making.”
Non-technical skills	3 (15.8%)	“Although AI should be used and developed to our advantage in medicine for efficiency, cost effectiveness and other huge benefits—this human connected and experience should never be forgotten.”
		“Communication skills are a pillar in the art of medicine and specialties dealing in sensitive matters will be less likely impacted by AI due to the fact that people will want other people to speak to about these.”
Optimistic	3 (15.8%)	“As long as humans continue to be involved in the end product (the provision of medical services), I do not fear the inclusion of AI in the process—if anything I welcome it.”
		“Interesting technology which I am sure will be a boon to medicine in the future”
Media reference	2 (10.5%)	“Most of my AI knowledge comes from Sam Harris”
		“Skynet doctor. Good and bad”

## Discussion

Most WA medical students surveyed were interested in AI and felt they have a basic understanding of what AI is. Despite this, few felt they understood the basic computational principles of AI and AI’s limitations. Students have identified that AI will likely impact medicine. The combination of student interest and perceived future impact, combined with self-reported lack of understanding suggests a deficit in the current medical curriculum in WA. These results are consistent with those observed internationally in previous studies.

While the public are supportive of the use of AI in healthcare, they are also wary about trusting AI systems [1]. Most Australian adults (61%) report a low level of understanding of AI and its uses [1, 3]. However, most Australian adults (86%) also want to know more about AI [1]. Previous surveys have suggested that an individual’s preconceptions about AI will change when further information, examples, and explanations are provided [3]. If AI becomes integrated into healthcare, it is likely that the public will expect doctors to be able to converse with them about the use of AI in their care.

The integration of AI into healthcare will undoubtably be complex. Future clinicians will need to understand AI and its role in healthcare. This will involve having a basic technical understanding and ability to critically evaluate new AI research and technologies. They will also be required to navigate complex ethical questions such as those involving algorithmic biases and transparency, as well as patient safety and privacy [18]. There is an opportunity to develop future clinician leaders with expertise in both AI and medicine [7, 21, 22].

Some medical schools are adapting. The recently opened Carle Illinois College of Medicine in Urbana-Champaign, Illinois, strongly integrates mathematics and data science into the medical curriculum [22]. However, medical student burnout is a recognised issue [23]. Integrating AI into an already crowded medical curriculum will be a challenge.

There are several limitations to this research. No previously validated survey instrument existed at the time of our study. As this is a voluntary survey there is also a high risk of self-selection bias, with students interested in AI potentially being more likely to complete the survey. The low response rate to this survey limits the generalisability of our results, however our response rate is comparable to previously published surveys of medical students’ attitudes towards AI [15].

## Conclusion

Medical students in WA appear to be interested in AI and believe that it will impact the practice of medicine. However, they have not received education on AI and do not feel they understand its basic computational principles or limitations. Most WA medical students are supportive of its introduction into the medical curriculum, and see AI as a tool and adjunct to existing clinical practice. AI education appears to be a current deficit in the medical curriculum in WA. These results are consistent with previous surveys conducted internationally.

## Supporting information

S1 Appendix Survey instrument. (PDF)

S1 Table Medical specialties most likely to be impacted by artificial intelligence, as ranked by medical students. (DOCX)

S2 Table Medical specialties least likely to be impacted by artificial intelligence, as ranked by medical students. (DOCX)

S1 Fig Impact of developments in AI on medical students’ feelings towards job security as a doctor. Percentages of students who were not at all concerned, concerned, neutral, not really concerned, not at all concerned with corresponding statement. Percentage ≤ 2 are not labelled in the figure to improve readability.
(TIF)

S2 Fig Impact of developments in AI on medical students’ liklihood of considering radiology as a career. Percentages of students who responded that they were much less liktely, less likely, neither, more likely, or much more to consider radiology as a career given developments in artificial intelligence. Percentage ≤ 2 are not labelled in the figure to improve readability.
(TIF)

S1 Dataset Minimal dataset. (CSV)
