# Supplementary material for: Western Australian medical students’ attitudes towards artificial intelligence in healthcare
Source: PLoS One. 2023 Aug 31;18(8):e0290642. doi: 10.1371/journal.pone.0290642 (PMC10470885; doi:10.1371/journal.pone.0290642)
Supplement: S1 Appendix — (PDF) [file pone.0290642.s001.pdf]

Western Australian Medical Students' Attitudes Towards Artificial Intelligence in Medicine

Start of Block: Default Question Block

Online Survey Participant Information

Western Australian Medical Students' Attitudes Towards Artificial Intelligence in Medicine

Project Team
Dr Jonathon Stewart
Ms Juan Lu
Dr Adrian Goudie
Dr Peter Sprivulis
Dr Gerry Fegan
Dr Nestor Gahungu
Prof Girish Dwivedi

Invitation:
You are invited to participate in a short survey assessing medical students' attitudes towards the use of artificial intelligence in medicine. You are being asked to take part in this project because you are a medical student in Western Australia.

Aim of the Study (What is the project about?)
This study aims to provide descriptive statistics regarding Western Australian medical students' attitudes towards the use of artificial intelligence in medicine.

What does participation involve?
Participation in this study involves completing an online survey. This will require you to click boxes corresponding to your answer. **This will take about 5 minutes.** At the end of a survey there will be a voluntary text box where you can provide further thoughts.

Voluntary Participation and Withdrawal from the Study
Participation in this survey is completely voluntary. You can withdraw from the study at any time, without giving an explanation. There are no consequences to withdrawing from this survey. If you withdraw after beginning the survey, any data you provide will be destroyed.

Your privacy
This survey is completely anonymous. Your participation in this study and any information you provide will be deidentified and treated in a confidential manner. Information from this project will be published but your name and identifying details will not be used in any publication arising out of the research. The collected data will be kept in a non-identifiable format, in a password protected computer for ten years before being securely destroyed.

Possible Benefits
Artificial intelligence is likely to impact the practice of medicine in the future. We believe it is beneficial to obtain quantitative data of medical students' attitudes towards its use. You will be contributing to a broader understanding of medical students' attitudes towards artificial intelligence. Results of this study will be published in an appropriate journal and medical students will be sent a link to view these results.

Possible Risks and Risk Management Plan
This is a short, voluntary, anonymous survey. There are no foreseeable risks associated with the research.

Contacts
If you would like to discuss any aspect of this study, please feel free to contact Jonathon Stewart by phone (04[redacted] or by email (Jonathon.Stewart@research.uwa.edu.au)

Approval to conduct this research has been provided by the University of Western Australia, in accordance with its ethics review and approval procedures. Any person considering participation in this research project, or agreeing to participate, may raise any questions or issues with the researchers at any time. In addition, any person not satisfied with the response of researchers may raise ethics issues or concerns, and may make any complaints about this research project by contacting the Human Ethics office at UWA on (08) 6488 4703 or by emailing to humanethics@uwa.edu.au. All research participants are entitled to retain a copy of any Participant Information Form and/or Participant Consent Form relating to this research project. If you would like a copy of this form emailed to you, please contact Jonathon.stewart@research.uwa.edu.au

Consent Statement
I have read the information provided and any questions I have asked have been answered to my satisfaction. I agree to participate in this research project, but withdrawal is not possible once data are submitted

I understand that all identifiable information that I provide is treated as confidential and will not be released by the investigator in any form that may identify me unless I have consented to this. The only exception to this principle of confidentiality is if this information is required by law to be released.

I consent to participate in this research project

- ○ Yes
- ○ No

Skip To: End of Survey If Online Survey Participant InformationWestern Australian Medical Students' Attitudes Towards Artif... = No

---

What is your age?

- ○ under 20
- ○ 20-29
- ○ 30-39
- ○ 40-49
- ○ 50 or over

What is your gender?

- ○ Male
- ○ Female
- ○ Non-binary
- ○ Prefer not to say

Is your degree undergraduate or postgraduate?

- ◯ Undergraduate
- ◯ Postgradute

Have you started clinical rotations?

- ◯ Yes
- ◯ No

Page Break

Please rank your level of agreement with the following statements.

	Strongly Disagree	Somewhat disagree	Neither agree nor disagree	Somewhat agree	Strongly agree
I am interested in artificial intelligence in general.	○	○	○	○	○
I have a basic understanding of what artificial intelligence is.	○	○	○	○	○
I have an understanding of the basic computational principles of artificial intelligence.	○	○	○	○	○
I have an understanding of the limitations of artificial intelligence.	○	○	○	○	○

Page Break

Artificial Intelligence (AI) systems try to mimic human intelligence by using complicated computer programs. This can include learning, recognizing patterns and making decisions.

Please rank your level of agreement with the following statements

	Strongly Disagree	Somewhat disagree	Neither agree nor disagree	Somewhat agree	Strongly agree
Artificial intelligence will play an important role in medicine	○	○	○	○	○
I would use artificial intelligence tools when I become a medical doctor.	○	○	○	○	○
Artificial intelligence will improve medicine in general.	○	○	○	○	○
Some medical specialties will be replaced by artificial intelligence during my lifetime.	○	○	○	○	○

Page Break

Please rank your level of agreement with the following statements

	Strongly Disagree	Somewhat disagree	Neither agree nor disagree	Somewhat agree	Strongly agree
I have received teaching in artificial intelligence.	○	○	○	○	○
Artificial intelligence education should be part of medical training.	○	○	○	○	○
At the end of my medical degree, I will have a better understanding of the methods used to assess medical AI algorithm performance.	○	○	○	○	○
Overall, at the end of my medical degree, I feel I will possess the knowledge needed to work with AI in routine clinical practice.	○	○	○	○	○
I would like to receive more teaching focusing on artificial intelligence in medicine	○	○	○	○	○

Please rank your level of agreement with the following statements

	Strongly Disagree	Somewhat disagree	Neither agree nor disagree	Somewhat agree	Strongly agree
I expect to be informed if a clinical device is using artificial intelligence	○	○	○	○	○
I expect artificial intelligence tools will have explanation of how they are working	○	○	○	○	○
I expect to be given a choice as to whether an artificial intelligence tool is to be used or not	○	○	○	○	○
I expect to be able to override the recommendations of an artificial intelligence tool.	○	○	○	○	○

Page Break

Please select which specialties you think will be **MOST** impacted by artificial intelligence (select up to 5)

- ☐ Addiction Medicine
- ☐ Anaesthesia
- ☐ Dermatology
- ☐ Emergency Medicine
- ☐ General Practice
- ☐ Intensive Care Medicine
- ☐ Medical Administration
- ☐ Obstetrics and Gynaecology
- ☐ Occupational and Environmental Medicine
- ☐ Ophthalmology
- ☐ Paediatric Medicine
- ☐ Pain Medicine
- ☐ Palliative care
- ☐ Pathology
- ☐ Physician (including cardiology, endocrinology, nephrology, neurology etc.)
- ☐ Psychiatry

- [ ] Public Health Medicine
- [ ] Radiation Oncology
- [ ] Radiology
- [ ] Rehabilitation Medicine
- [ ] Sexual Health Medicine
- [ ] Sports and exercise medicine
- [ ] Surgery (including general surgery, cardiothoracic surgery, plastic surgery etc.)

Page Break

Please select which specialties you think will be **LEAST** impacted by artificial intelligence (select up to 5)

- ☐ Addiction Medicine
- ☐ Anaesthesia
- ☐ Dermatology
- ☐ Emergency Medicine
- ☐ General Practice
- ☐ Intensive Care Medicine
- ☐ Medical Administration
- ☐ Obstetrics and Gynaecology
- ☐ Occupational and Environmental Medicine
- ☐ Ophthalmology
- ☐ Paediatric Medicine
- ☐ Pain Medicine
- ☐ Palliative care
- ☐ Pathology
- ☐ Physician (including cardiology, endocrinology, nephrology, neurology etc.)
- ☐ Psychiatry

- [ ] Public Health Medicine
- [ ] Radiation Oncology
- [ ] Radiology
- [ ] Rehabilitation Medicine
- [ ] Sexual Health Medicine
- [ ] Sports and exercise medicine
- [ ] Surgery (including general surgery, cardiothoracic surgery, plastic surgery etc.)

Page Break

Regarding job security as a doctor, developments in artificial intelligence make me feel

- ○ Very concerned
- ○ Concerned
- ○ Neutral
- ○ Not really concerned
- ○ Not at all concerned

Please provide brief explanation why you feel that way. [Optional]

____

____

____

____

____

Page Break

Given developments in artificial intelligence, how likely are you to consider a career in radiology?

- ○ Much less likely
- ○ Less likely
- ○ Neither
- ○ More likely
- ○ Much more Likely

Please provide brief explanation why. [Optional]

____

____

____

____

____

Page Break

Please select all of the following that you have heard of

- ☐ Machine learning
- ☐ Deep learning
- ☐ Convolutional Neural Networks
- ☐ Generative Adversarial Neural Networks
- ☐ ImageNet
- ☐ Deepfakes
- ☐ DeepMind
- ☐ OpenAI
- ☐ Python
- ☐ TensorFlow
- ☐ PyTorch
- ☐ GPT-3

Page Break

If you have any further thoughts or comments about artificial intelligence in medicine please write them in the text box below.

____

____

____

____

____

End of Block: Default Question Block
