# Supplementary material for: Western Australian medical students’ attitudes towards artificial intelligence in healthcare
Source: PLoS One. 2023 Aug 31;18(8):e0290642. doi: 10.1371/journal.pone.0290642 (PMC10470885; doi:10.1371/journal.pone.0290642)
Supplement: S1 Table — (DOCX) [file pone.0290642.s002.docx]

| **Specialty** | **Count** | **Percent** |
| --- | --- | --- |
| Radiology | 96 | 72% |
| Pathology | 78 | 58% |
| Medical Administration | 60 | 45% |
| Surgery (including general surgery, cardiothoracic surgery, plastic surgery etc.) | 47 | 35% |
| Anaesthesia | 42 | 31% |
| Dermatology | 37 | 28% |
| General Practice | 32 | 24% |
| Radiation Oncology | 32 | 24% |
| Intensive Care Medicine | 29 | 22% |
| Ophthalmology | 26 | 19% |
| Public Health Medicine | 24 | 18% |
| Emergency Medicine | 23 | 17% |
| Occupational and Environmental Medicine | 14 | 10% |
| Physician (including cardiology, endocrinology, nephrology, neurology etc.) | 10 | 7% |
| Sports and exercise medicine | 7 | 5% |
| Pain Medicine | 6 | 4% |
| Obstetrics and Gynaecology | 5 | 4% |
| Palliative care | 4 | 3% |
| Rehabilitation Medicine | 4 | 3% |
| Sexual Health Medicine | 4 | 3% |
| Addiction Medicine | 2 | 1% |
| Psychiatry | 2 | 1% |
| Paediatric Medicine | 1 | 1% |
