# Supplementary material for: Western Australian medical students’ attitudes towards artificial intelligence in healthcare
Source: PLoS One. 2023 Aug 31;18(8):e0290642. doi: 10.1371/journal.pone.0290642 (PMC10470885; doi:10.1371/journal.pone.0290642)
Supplement: S2 Table — (DOCX) [file pone.0290642.s003.docx]

| **Specialty** | **Count** | **Percent** |
| --- | --- | --- |
| Psychiatry | 82 | 61% |
| Palliative care | 65 | 49% |
| Obstetrics and Gynaecology | 55 | 41% |
| General Practice | 49 | 37% |
| Paediatric Medicine | 45 | 34% |
| Emergency Medicine | 43 | 32% |
| Addiction Medicine | 38 | 28% |
| Sexual Health Medicine | 33 | 25% |
| Physician (including cardiology, endocrinology, nephrology, neurology etc.) | 30 | 22% |
| Pain Medicine | 28 | 21% |
| Rehabilitation Medicine | 27 | 20% |
| Surgery (including general surgery, cardiothoracic surgery, plastic surgery etc.) | 27 | 20% |
| Intensive Care Medicine | 26 | 19% |
| Sports and exercise medicine | 21 | 16% |
| Anaesthesia | 12 | 9% |
| Public Health Medicine | 10 | 7% |
| Medical Administration | 8 | 6% |
| Occupational and Environmental Medicine | 7 | 5% |
| Ophthalmology | 6 | 4% |
| Dermatology | 5 | 4% |
| Pathology | 3 | 2% |
| Radiology | 3 | 2% |
| Radiation Oncology | 2 | 1% |
