# Supplementary figures and images for: Western Australian medical students’ attitudes towards artificial intelligence in healthcare
Source: PLoS One. 2023 Aug 31;18(8):e0290642. doi: 10.1371/journal.pone.0290642 (PMC10470885; doi:10.1371/journal.pone.0290642)

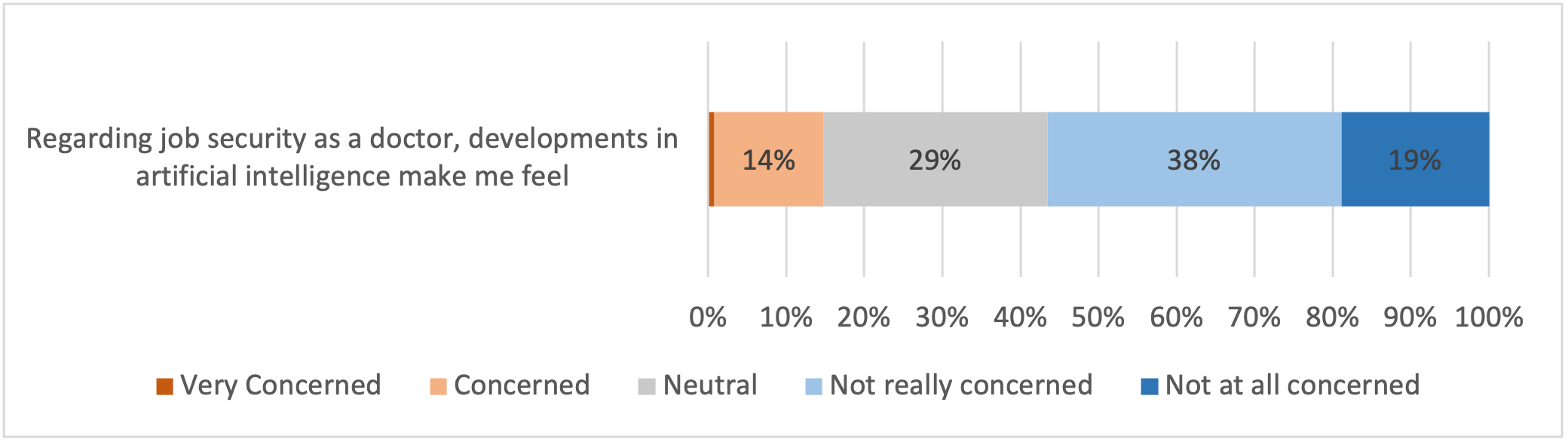

Supplement: S1 Fig — Percentages of students who were not at all concerned, concerned, neutral, not really concerned, not at all concerned with corresponding statement. Percentage ≤ 2 are not labelled in the figure to improve readability. (TIF) [file pone.0290642.s004.tif]

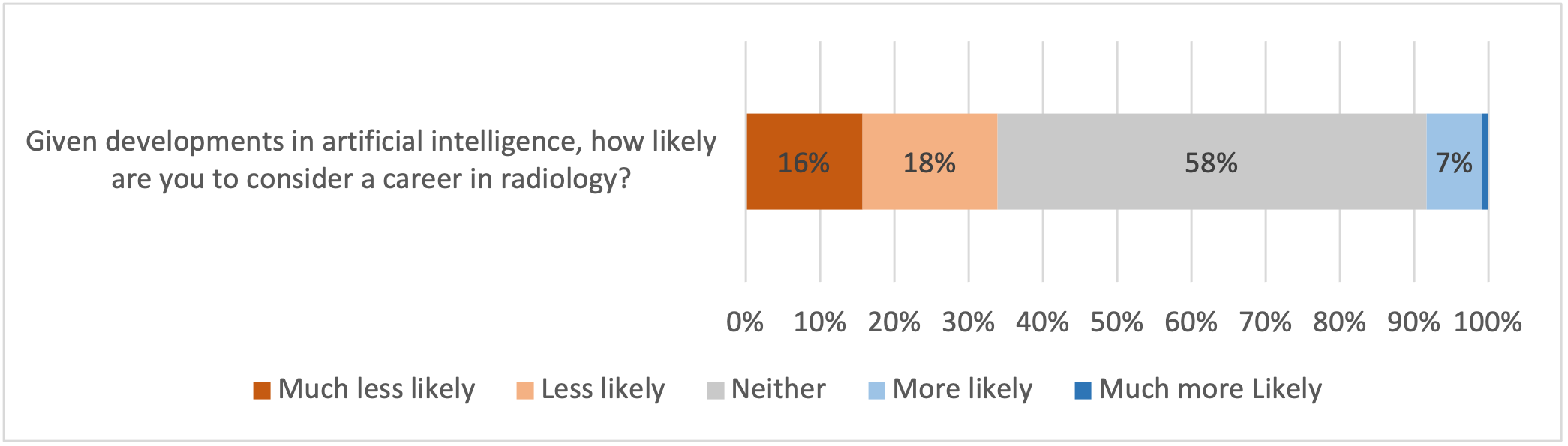

Supplement: S2 Fig — Percentages of students who responded that they were much less liktely, less likely, neither, more likely, or much more to consider radiology as a career given developments in artificial intelligence. Percentage ≤ 2 are not labelled in the figure to improve readability. (TIF) [file pone.0290642.s005.tif]
